# Resistance exercise initiates mechanistic target of rapamycin (mTOR) translocation and protein complex co-localisation in human skeletal muscle

**DOI:** 10.1038/s41598-017-05483-x

**Published:** 2017-07-10

**Authors:** Zhe Song, Daniel R. Moore, Nathan Hodson, Carl Ward, Jessica R. Dent, Mary F. O’Leary, Andrew M. Shaw, D. Lee Hamilton, Sovan Sarkar, Yann-Gaël Gangloff, Troy A. Hornberger, Lawrence L. Spriet, George J. Heigenhauser, Andrew Philp

**Affiliations:** 1 School of Sport, Exercise and Rehabilitation Sciences, University of Birmingham, England, UK; 2 Faculty of Kinesiology and Physical Education, University of Toronto, Toronto, Canada; 3 Human Health and Nutritional Sciences, University of Guelph, Guelph, Canada; 4 Institute of Cancer and Genomic Sciences, University of Birmingham, England, UK; 5 Institute of Inflammation and Ageing, University of Birmingham, England, UK; 6 School of Sport, University of Stirling, Scotland, UK; 7 Institut NeuroMyoGene (INMG), University Lyon 1, INSERM U 1217, Lyon, France; 8 Department of Comparative Biosciences, The University of Wisconsin, Madison, USA; 9 Department of Medicine, McMaster University, Hamilton, Canada

## Abstract

The mechanistic target of rapamycin (mTOR) is a central mediator of protein synthesis in skeletal muscle. We utilized immunofluorescence approaches to study mTOR cellular distribution and protein-protein co-localisation in human skeletal muscle in the basal state as well as immediately, 1 and 3 h after an acute bout of resistance exercise in a fed (FED; 20 g Protein/40 g carbohydrate/1 g fat) or energy-free control (CON) state. mTOR and the lysosomal protein LAMP2 were highly co-localised in basal samples. Resistance exercise resulted in rapid translocation of mTOR/LAMP2 towards the cell membrane. Concurrently, resistance exercise led to the dissociation of TSC2 from Rheb and increased in the co-localisation of mTOR and Rheb post exercise in both FED and CON. In addition, mTOR co-localised with Eukaryotic translation initiation factor 3 subunit F (eIF3F) at the cell membrane post-exercise in both groups, with the response significantly greater at 1 h of recovery in the FED compared to CON. Collectively our data demonstrate that cellular trafficking of mTOR occurs in human muscle in response to an anabolic stimulus, events that appear to be primarily influenced by muscle contraction. The translocation and association of mTOR with positive regulators (i.e. Rheb and eIF3F) is consistent with an enhanced mRNA translational capacity after resistance exercise.

## Introduction

Resistance training is an effective strategy to increase muscle strength and muscle hypertrophy, with the latter ultimately mediated by an exercise-induced increase in muscle protein synthesis and net protein balance^1^. Skeletal muscle protein balance is generally dependent on the activity of the serine/threonine protein kinase mechanistic target of rapamycin (mTOR), which when active stimulates protein synthesis and attenuates protein degradation^2^. mTOR exists as 1 of 2 complexes (mTORC1 and mTORC2), with their respective substrate preference and, ultimately, biological activity related to the specific associated subunits^2^. For example, mTORC1 contains mTOR, GβL, raptor, DEPTOR and PRAS40 and is inhibited by the bacterial macrolide rapamycin^2^. In contrast, mTORC2 consists of mTOR, rictor, GβL, Sin1, DEPTOR and Protor/PRR5 and is insensitive to acute rapamycin administration^2^. Although each mTOR complex responds to unique subsets of biological stimuli and generally localize to different subcellular compartments^3^, mTORC1 is the most widely studied of the two complexes and responds to anabolic stimuli such as insulin, amino acids, and/or resistance exercise^2^. For example, acute administration of rapamycin blocks the independent anabolic effects of resistance exercise and amino acid ingestion on mTOR signaling molecule phosphorylation and subsequently protein synthesis in human skeletal muscle^4, 5^. Collectively, these data highlight a pivotal role for mTORC1 activity in the regulation of muscle protein synthesis in response to resistance exercise and amino acid ingestion.

Current understanding regarding the physiological regulation of mTOR in human skeletal muscle has resulted in large part from phosphorylation-specific profiling of the mTOR pathway in response to anabolic stimuli^6^. Consistent with the ability of resistance exercise to increase muscle protein synthesis in the fasted state^7, 8^, phosphorylation of mTOR substrates (as a proxy for mTOR activity) has indicated that mTOR is activated following resistance exercise^9^, with this response maintained for at least 24 h post-exercise^10^. Moreover, the provision of exogenous amino acids (either orally or intravenously) augments post-exercise rates of muscle protein synthesis^11, 12^, which is generally coincident with changes in phosphorylation status of proteins within the mTOR signaling cascade that are consistent with enhanced translational activity^13, 14^.

Beyond immunoblotting approaches, *in vitro* studies have indicated that cellular localization and protein-protein interaction may be fundamentally important in the regulation of mTOR activity in response to physiological stimuli^3^. Following mitogen or amino acid stimulation *in vitro*, mTOR has been observed to translocate to the lysosome where it associates with GTP bound ras-homolog enriched in brain (Rheb) to achieve full activation^15^. In contrast, amino acid removal results in mTOR dissociation from the Rag GTPases and hence the lysosome, subsequently rendering it inactive^15^. mTOR interaction with Rheb is restricted in basal conditions due to the enzymatic activity of the tuberous sclerosis complex proteins TSC1 and TSC2, which maintain Rheb in a GDP-bound state^16^. However, phosphorylation of TSC2 by AKT leads to TSC2 inactivation^17^, Rheb-TSC2 dissociation, GTP-loading of Rheb and interaction with mTOR^18^. Jacobs and coworkers recently reported that mTOR and TSC2 associate at the lysosome in mouse tibialis-anterior skeletal muscle, with eccentric contractions stimulating an increase in mTOR-lysosomal localisation and subsequent dissociation of TSC2 from the lysosome^19^. Collectively these studies would suggest that targeting of mTOR to the lysosome is a fundamentally important event to initiate cellular protein synthesis^20^.

The goal of the present study was to examine cellular distribution and co-localization of proteins involved in mTOR complex assembly/activity in human skeletal muscle in response to resistance exercise. In addition, we also evaluated the effect of post-exercise protein/carbohydrate ingestion on mTOR complex assembly and localisation. We hypothesized that resistance exercise would increase mTOR abundance at the lysosomal surface to facilitate interaction with Rheb and that post-exercise nutrients would augment these responses.

## Results

### Determining mTOR, Rheb, TSC2 and eIF3F antibody specificity in human skeletal muscle

**Figure 1:**
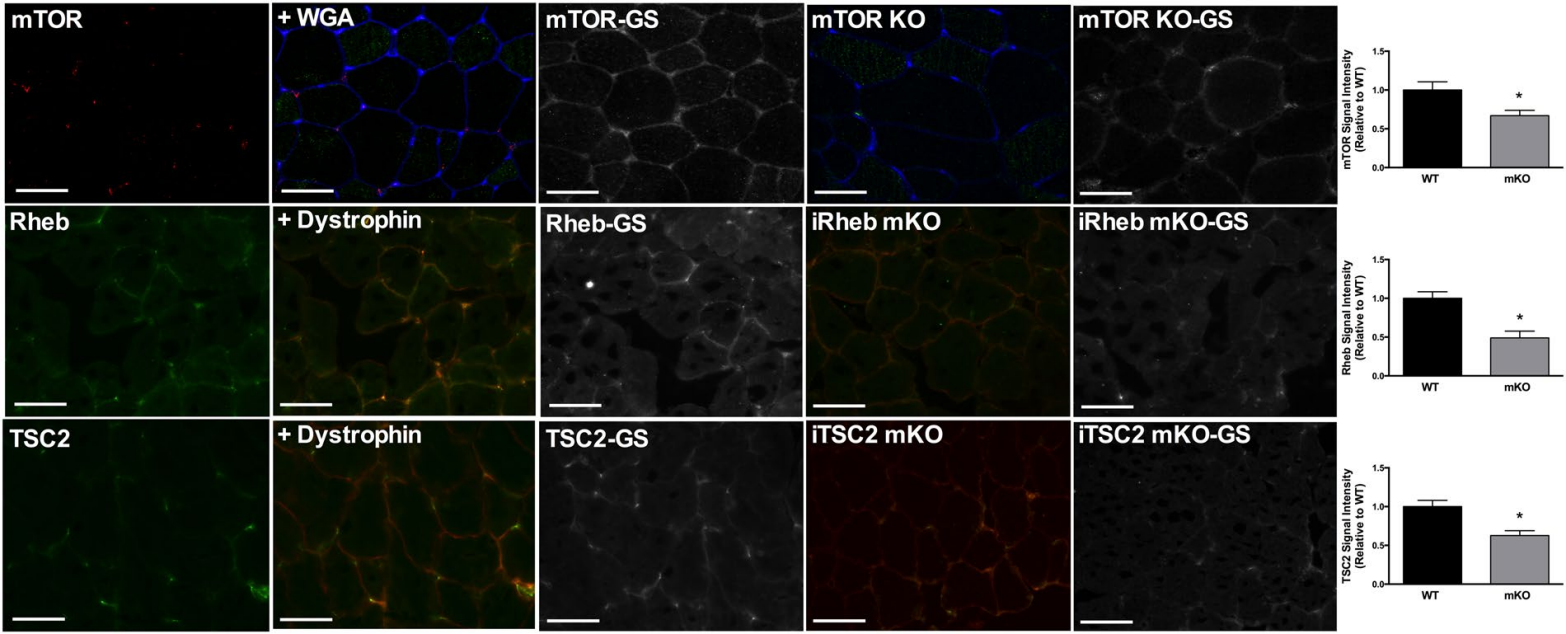
mTOR, Rheb and TSC2 antibody validation. Immunofluorescence labeling of endogenous proteins was performed as described in the Methods section. Cross-sections were stained with the antibodies relating to mTOR, Rheb and TSC2 in wild-type mice (WT – Left panel) or in muscle from mTOR, Rheb and TSC2 knockout mice (mKO). Regionality of staining is provided through co-staining with WGA or Dystrophin as a marker of the cell membrane. All image processing was kept constant between images, including exposure time, gain, and image frame. GS – Grey scale image *Significantly different to WT (*P* < 0.05).

**Figure 2:**
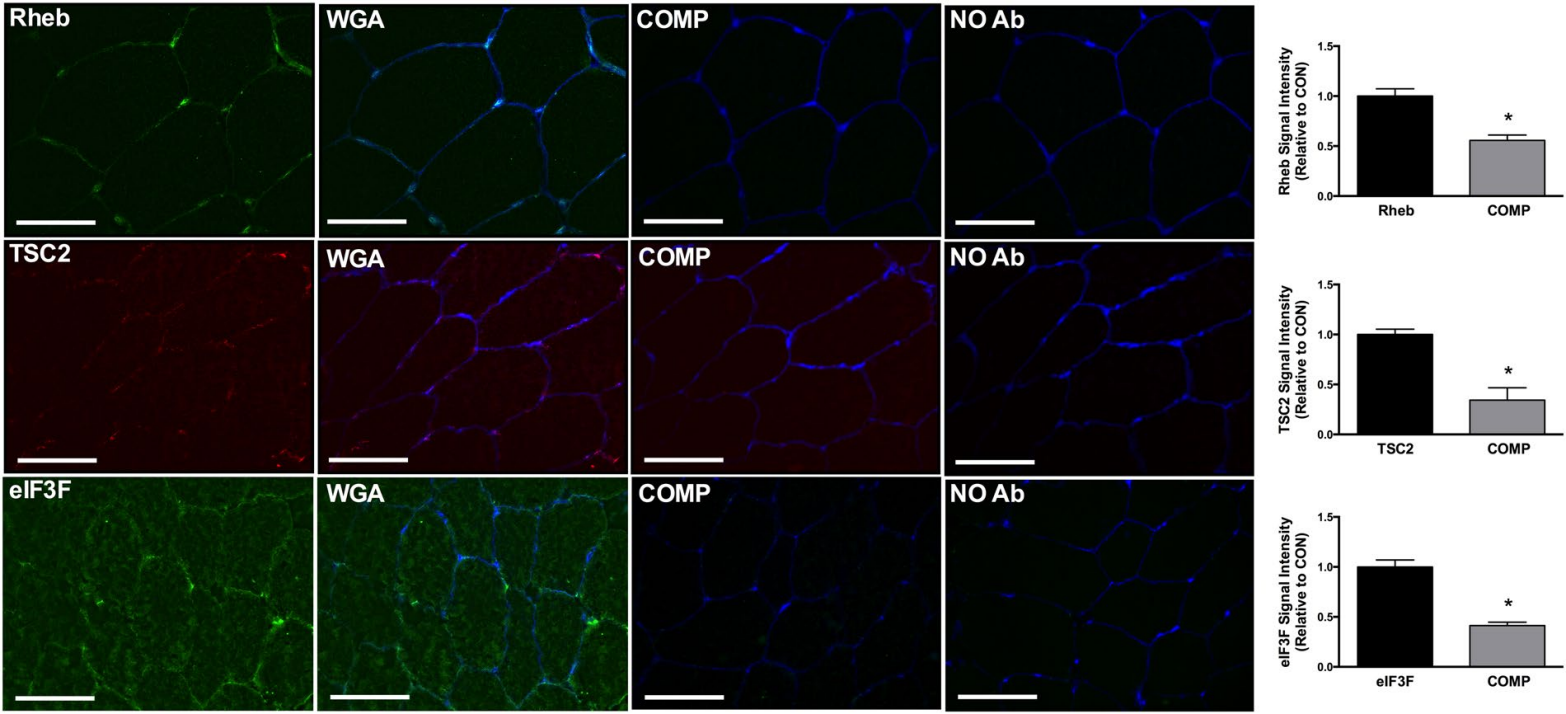
Rheb, TSC2 and eIF3F peptide competition assays. Immunofluorescence labeling of endogenous proteins was performed in human basal samples (CON – Left panel) as described in the Methods section. Regionality of staining is provided through co-staining with WGA or Dystrophin as a marker of the cell membrane. Competition (COMP) assays were performed to demonstrate antibody specificity of TSC2, eIF3F and Rheb. Non-specific binding/antibody efficiency was examined through imaging samples in which secondary antibody was used without prior primary antibody incubation (No Ab). All image processing was kept constant between images, including exposure time, gain, and image frame. *Significantly different to CON (*P* < 0.05).

To determine the specificity of antibodies towards mTOR and mTOR-associated proteins, we performed a number of validation approaches. To test the specificity of the mTOR, Rheb and TSC2 antibody, labelling experiments were performed in skeletal muscle from wild-type vs. whole-body mTOR knock-out^21^ and muscle-specific, tamoxifen-inducible Rheb and TSC2 mouse^22^ models (Fig. 1). For each protein, sensitivity to the endogenous protein was evident through a ~50% loss of signal in the KO/mKO sample (Fig. 1), whilst the remaining signal could be attributed to non-specific binding or heightened background due to species cross-reactivity. To test the antibodies specifically in human skeletal muscle, further validation was then performed using peptide competition assays (Rheb/eIF3F), or using the recombinant protein used in the antibody synthesis (TSC2) (Fig. 2). Finally, we examined non-specific binding of each antibody through omitting the primary antibody (Fig. 2). In each case we demonstrate that the antibodies used show specificity to their target protein (Figs 1 and 2).

### Synergistic effect of resistance exercise and post-exercise nutrition on S6K1 and AKT activity

**Figure 3:**
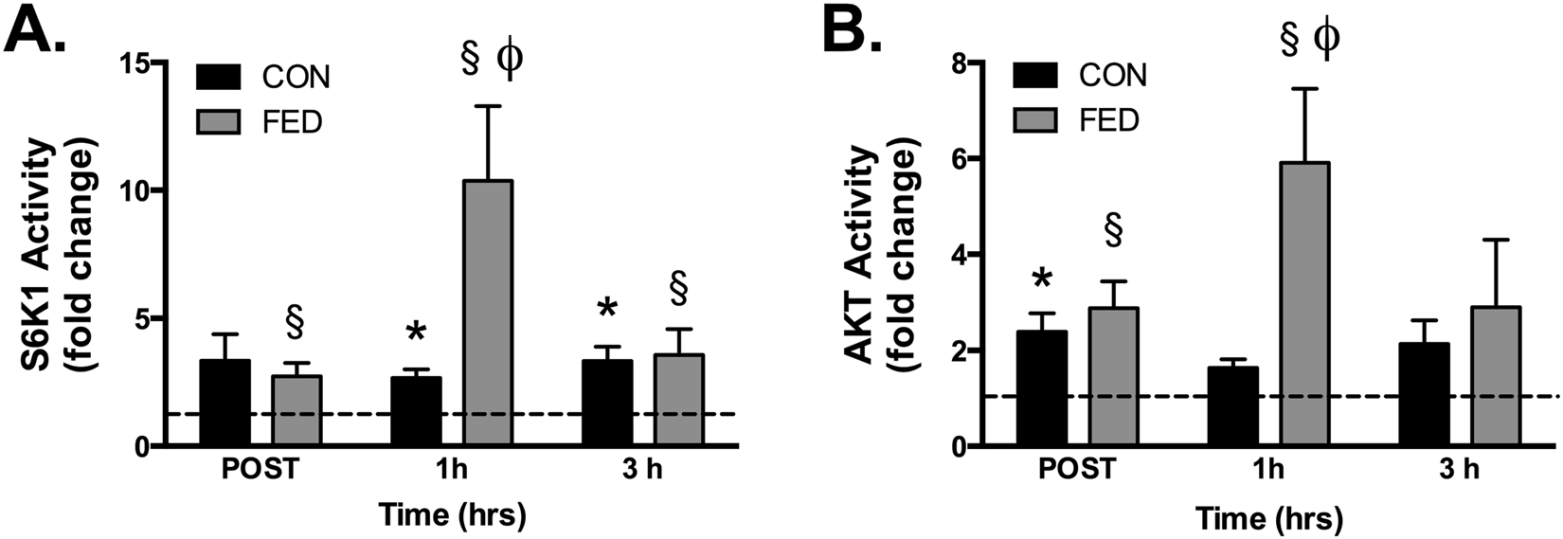
Post-exercise activation of S6K1 and AKT are augmented by PRO/CHO ingestion. Resistance exercise increased S6K1 activity, with this response significantly higher in the FED group 1 h post exercise (**A**). In parallel, AKT kinase activity was increased post exercise, with this response significantly higher in the FED group 1 h post exercise (**B**). Data presented as mean ± SEM (n = 7/group). *Significantly different to PRE-CON, ^§^Significantly different to PRE-FED, ^Φ^Significantly different to 1h-CON (*P* < 0.05).

S6K1 activity was rapidly increased post-exercise in both groups, with this response augmented in the FED group 1 h post exercise compared to control (CON: 3-fold; FED: 10-fold). In parallel, AKT kinase activity was rapidly increased post exercise in both groups, with this response significantly higher in the FED group 1 h post exercise (CON: 1-fold; FED: 6-fold)(Fig. 3).

### mTOR/LAMP2 co-localisation and cellular distribution in basal and exercise-stimulated human skeletal muscle

**Figure 4:**
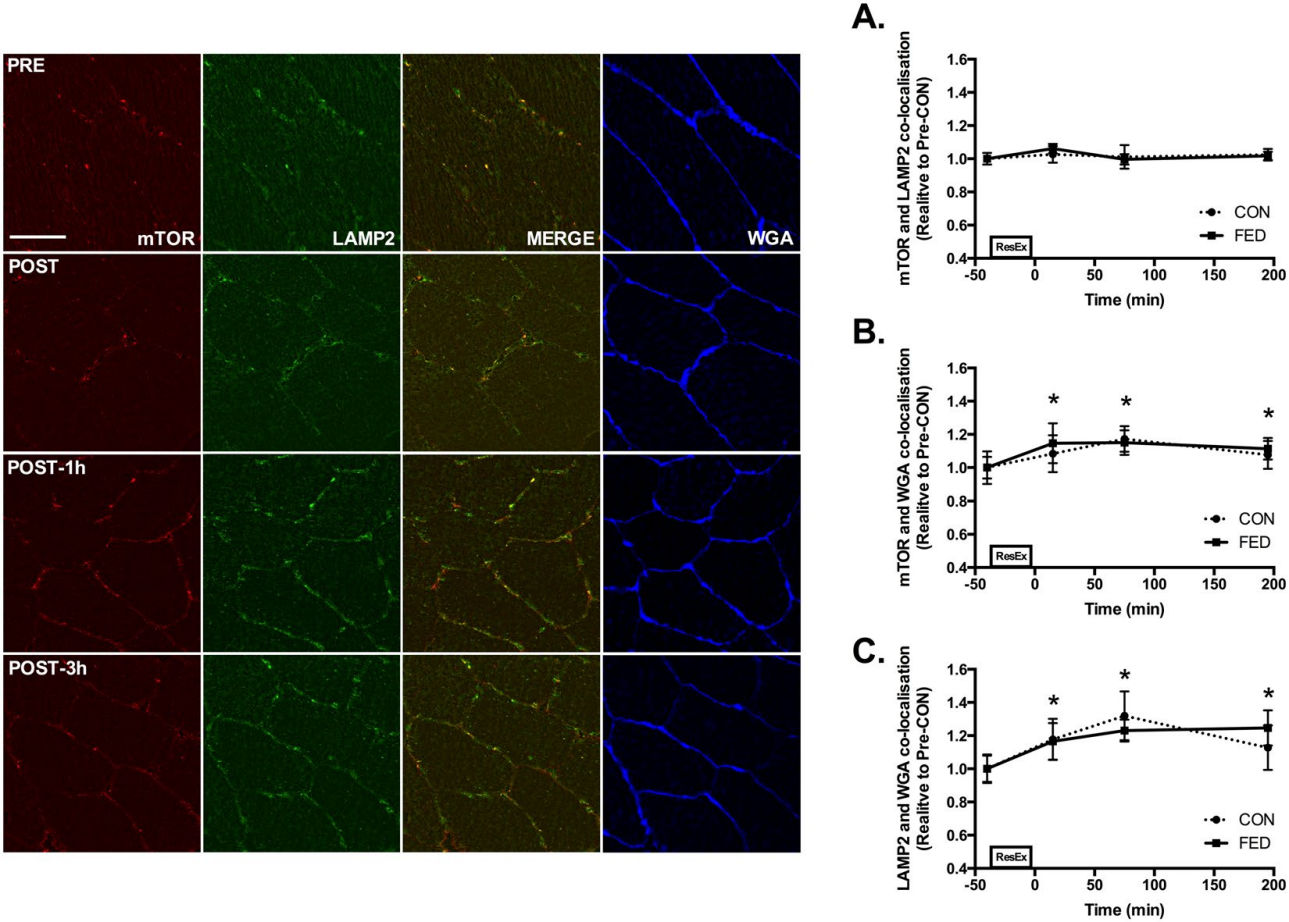
mTOR/LAMP2 complex translocation to the cell membrane following resistance exercise. Representative image of mTOR (Red), LAMP2 (Green), composite image of mTOR and LAMP2 (Merge) and WGA (Blue) for one subject from CON across the experimental time course. Yellow regions represent mTOR and LAMP2 interaction. Immunofluorescence quantification of mTOR and LAMP2 Co-localization (**A**), quantification of mTOR associated with the cell membrane marker WGA (**B**) and quantification of LAMP2 associated with WGA (**C**). Circles represent CON, squares represent FED. All image processing was kept constant between images, including exposure time, gain, and image frame. All data presented relative to the Pre exercise CON. Data presented as mean ± SEM (n = 7/group). *Significantly different to PRE-CON (*P* < 0.05).

**Figure 5:**
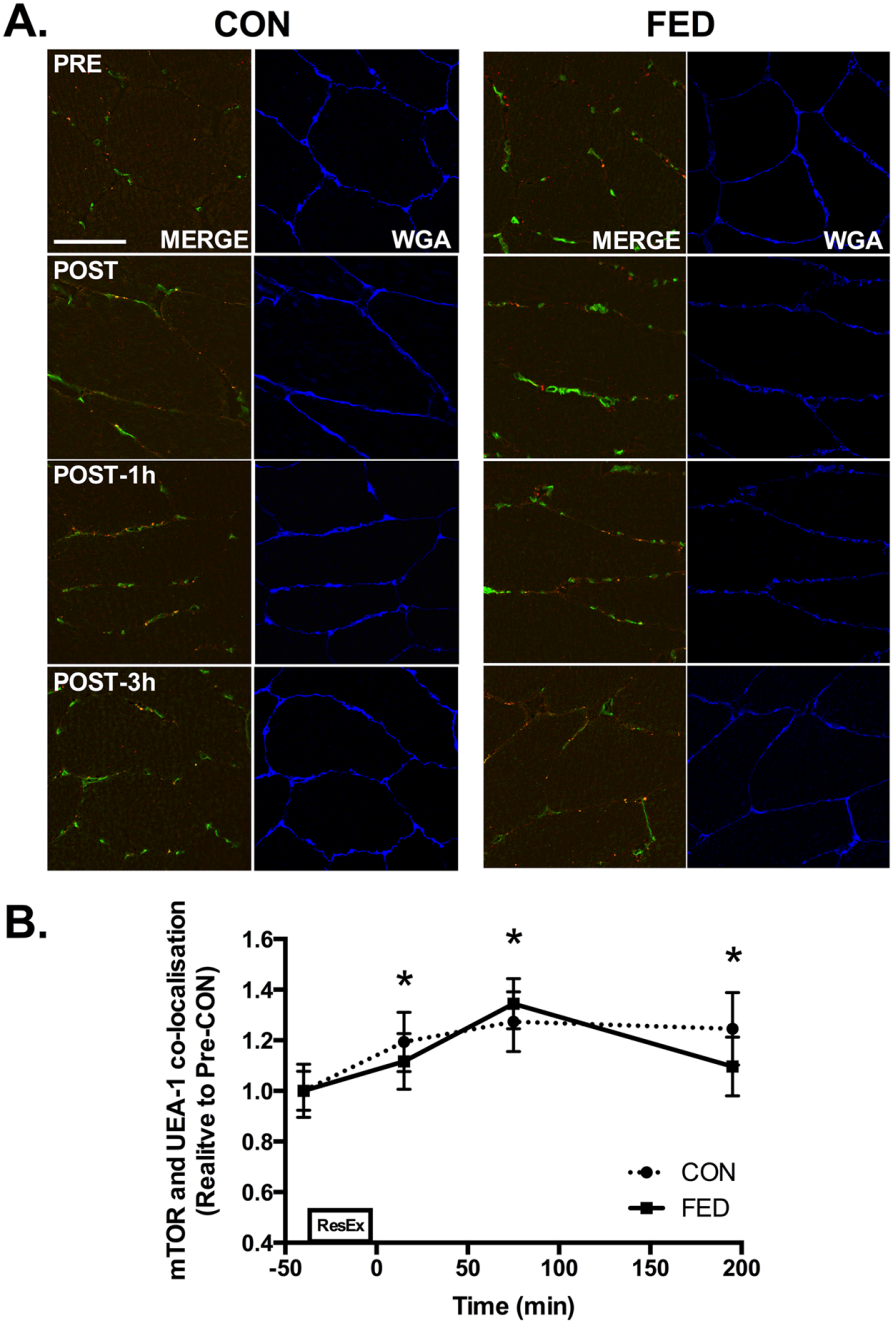
mTOR translocates to the microvasculature following resistance exercise. Immunofluorescence quantification of mTOR (Red) and UEA-I (Green) interaction, displayed as a composite image (Merge) and WGA (Blue). Yellow regions represent mTOR and UEA-I interaction. Each panel represents one subject from CON and FED across the experimental time course (**A**). Group data is quantified and reported in (**B**). Circles represent CON, squares represent FED. All image processing was kept constant between images, including exposure time, gain, and image frame. All data presented relative to the Pre exercise CON. Data presented as mean ± SEM (n = 7/group). *Significantly different to PRE-CON (*P* < 0.05).

The total amount of mTOR did not change during the experimental time course in either the CON or FED groups (data not shown). mTOR and LAMP2 were found to be highly colocalised in human skeletal muscle in both basal and exercise stimulated conditions suggesting that mTOR is located in close proximity to the late-lysosome in basal skeletal muscle (Fig. 4A). In both CON and FED we observed relocation of mTOR from cytosolic regions to the sarcolemmal membrane following resistance exercise as quantified by mTOR localisation with WGA positive regions, with a ~20% increase in membrane associated mTOR observed across all post-exercise time points (Fig. 4B). In parallel, we observed relocation of LAMP2 from cytosolic regions to the sarcolemmal membrane following resistance exercise as quantified by LAMP2 localisation with WGA positive regions, with a ~20% increase in membrane associated LAMP2 observed across all post-exercise time points (Fig. 4C). To examine the potential physiological relevance of mTOR translocation to the plasma membrane, we examined mTOR co-localisation with nuclei and blood vessels. Whilst mTOR did not associate with the nucleus post exercise (DAPI positive regions - data not shown), we did observe mTOR accumulating in regions close to the blood capillary endothelium as determined via UEA-I Lectin positive staining (Fig. 5).

### TSC2 dissociates from Rheb and plasma membrane regions post-exercise

**Figure 6:**
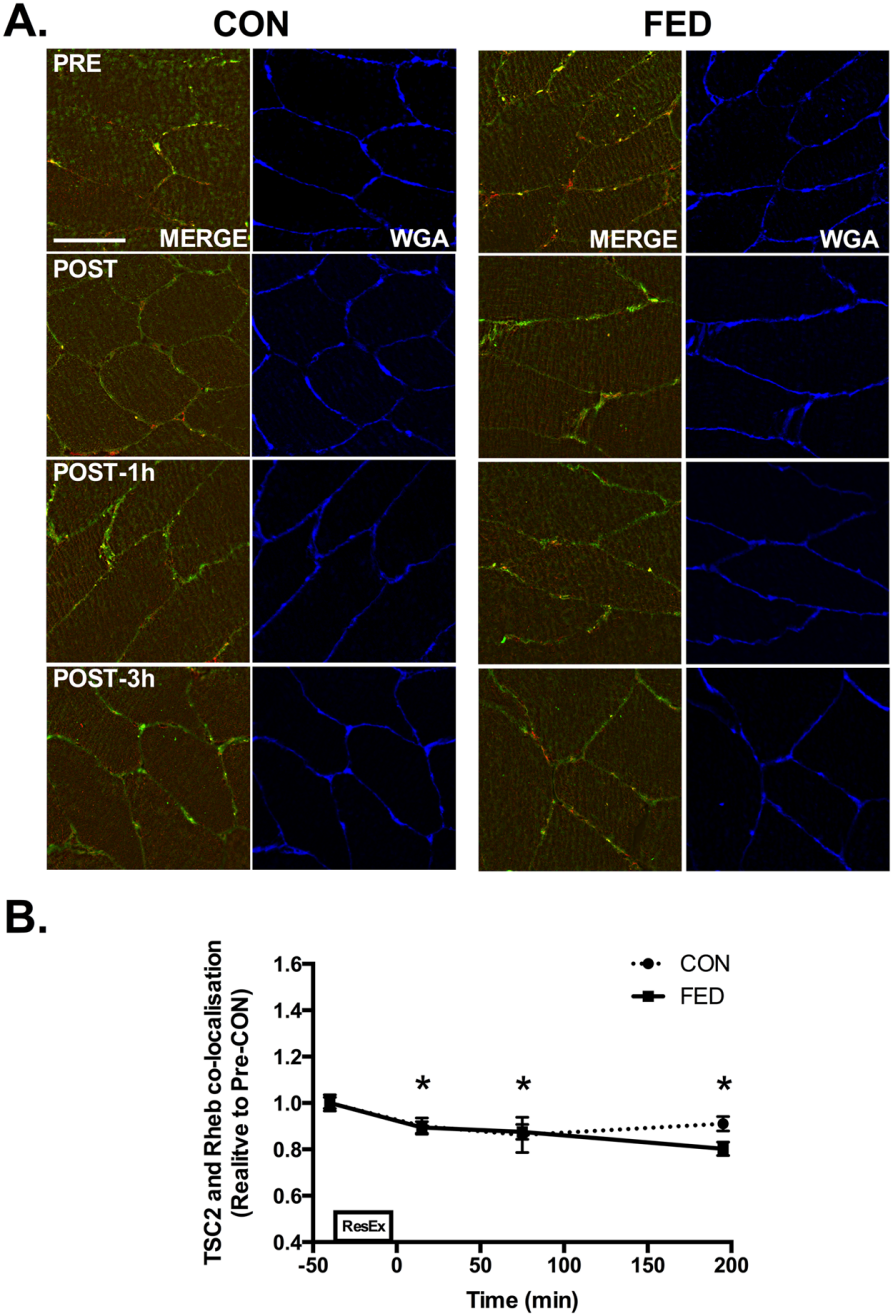
TSC2 and Rheb dissociate following resistance exercise. Immunofluorescence quantification of TSC2 (Red) and Rheb (Green) interaction, displayed as a composite image (Merge) and WGA (Blue). Yellow regions represent TSC2 and Rheb interaction. Each panel represents one subject from CON and FED across the experimental time course (**A**). Group data is quantified and reported in (**B**). Circles represent CON, squares represent FED. All image processing was kept constant between images, including exposure time, gain, and image frame. All data presented relative to the Pre exercise CON. Data presented as mean ± SEM (n = 7/group). *Significantly different to PRE-CON (*P* < 0.05).

**Figure 7:**
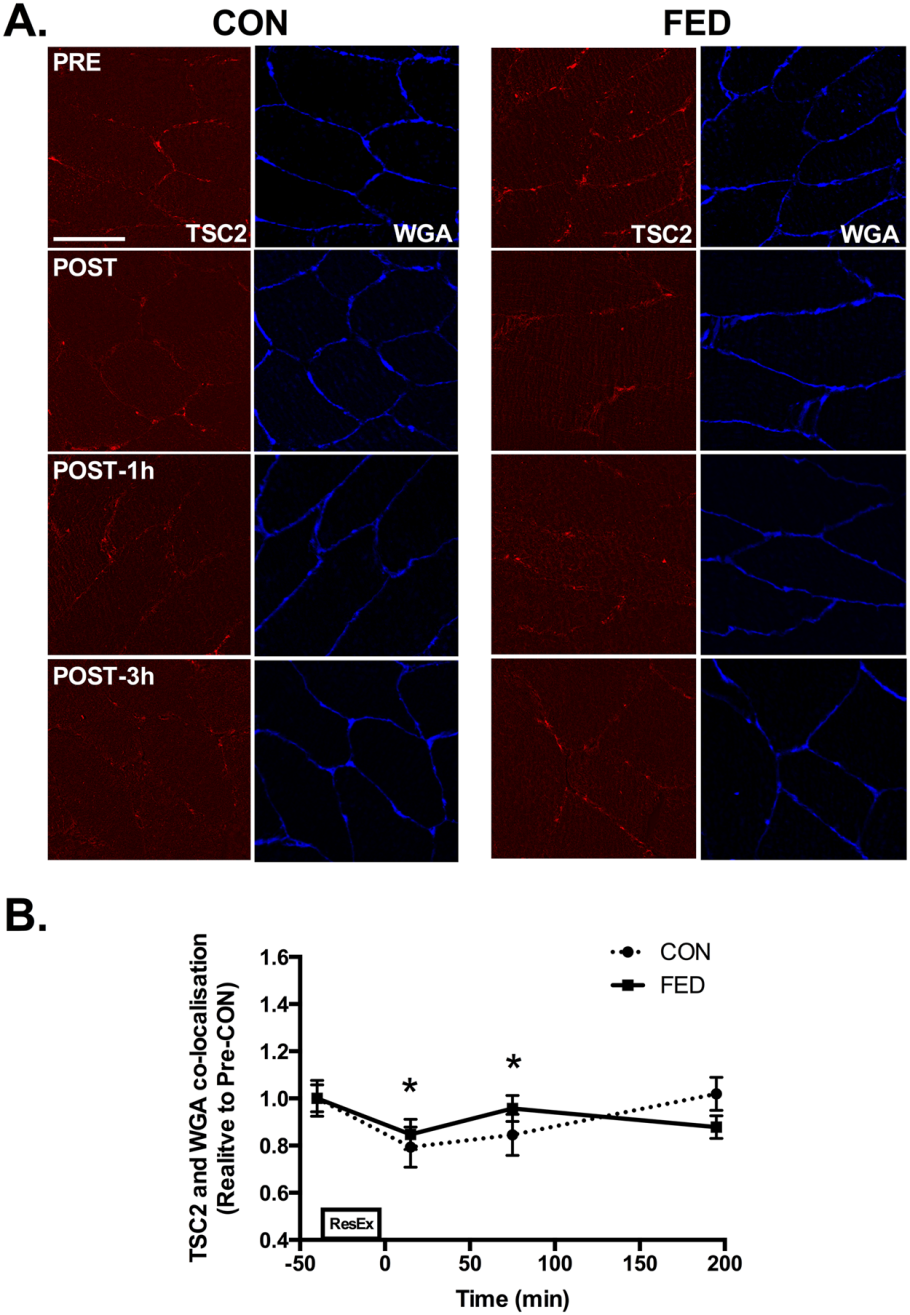
TSC2 dissociates from the plasma membrane following resistance exercise. Immunofluorescence quantification of TSC2 (Red) and the plasma membrane marker WGA (Blue). Each panel represents one subject from CON and FED across the experimental time course (**A**). Group data is quantified and reported in (**B**). Circles represent CON, squares represent FED. All image processing was kept constant between images, including exposure time, gain, and image frame. All data presented relative to the Pre exercise CON. Data presented as mean ± SEM (n = 7/group). *Significantly different to PRE-CON (*P* < 0.05).

TSC2 and Rheb were found to associate in close proximity to the plasma membrane in pre-exercise samples in both the CON and FED groups (Fig. 6). Following contraction, TSC2 and Rheb association decreased by ~20% across the entire post-exercise recovery period in both the CON and FED groups (Fig. 6). The dissociation of TSC2 and Rheb post exercise appeared to be mediated by TSC2 export from the cell membrane as we observed a ~30% decrease in TSC2 association with the plasma membrane (Fig. 7) post exercise in both the CON and FED groups.

### mTOR co-localises with Rheb and eIF3f post-exercise

**Figure 8:**
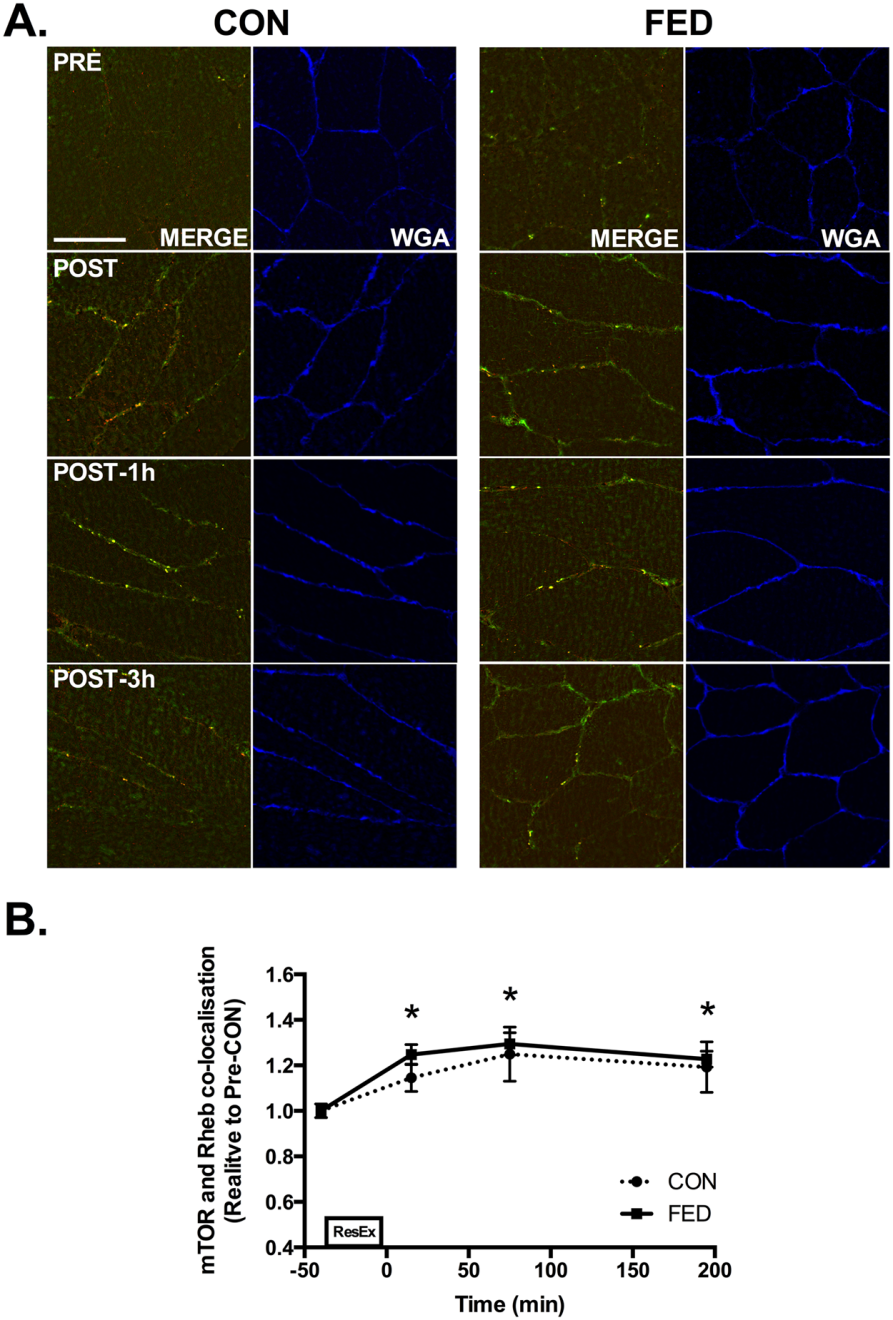
Resistance exercise increases mTOR and Rheb co-localisation at the plasma membrane. Immunofluorescence quantification of mTOR (Red) and Rheb (Green) interaction, displayed as a composite image (Merge) and WGA (Blue). Yellow regions represent mTOR and Rheb interaction. Each panel represents one subject from CON and FED across the experimental time course (**A**) Group data is quantified and reported in (**B**) Circles represent CON, squares represent FED. All image processing was kept constant between images, including exposure time, gain, and image frame. All data presented relative to the Pre exercise CON. Data presented as mean ± SEM (n = 7/group). *Significantly different to PRE-CON (*P* < 0.05).

**Figure 9:**
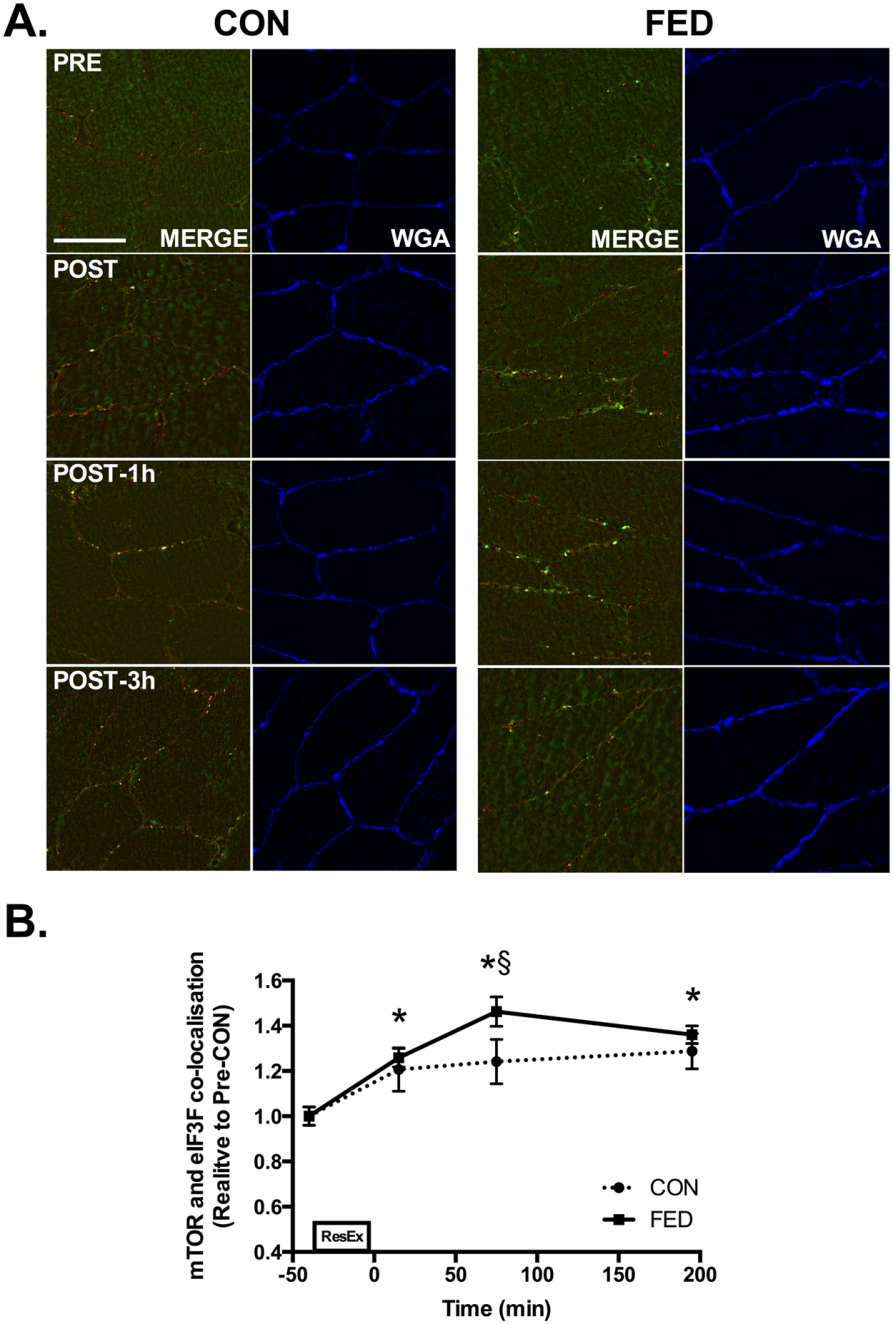
Resistance exercise increases mTOR and eIF3F co-localisation at the plasma membrane. Immunofluorescence quantification of mTOR (Red) and eIF3F (Green) interaction, displayed as a composite image (Merge) and WGA (Blue). Yellow regions represent mTOR and eIF3F interaction. Each panel represents one subject from CON and FED across the experimental time course (**A**). Group data is quantified and reported in (**B**). Circles represent CON, squares represent FED. All image processing was kept constant between images, including exposure time, gain, and image frame. All data presented relative to the Pre exercise CON. Data presented as mean ± SEM (n = 7/group). *Significantly different to PRE-CON (*P* < 0.05). ^§^Significantly different between groups (*P* < 0.05).

Given that mTOR/LAMP2 complexes were observed to re-locate to the plasma membrane post-exercise, we next examined whether mTOR redistribution might facilitate association with proteins implicated in mTOR activity. The most characterised positive regulator of mTOR is the GTPase Rheb. We observed a ~30% increase in mTOR and Rheb co-localisation post exercise in both the CON and FED groups (Fig. 8). To examine whether mTOR translocation to the membrane facilitated interaction with factors associated with protein synthesis we examined mTOR and eIF3F co-localisation. Resistance exercise resulted in rapid and sustained increases in mTOR association with eIF3F in both the CON and FED groups. Interestingly, the magnitude of association between mTOR and eIF3F was ~20% greater in the FED group 1 h post exercise compared to CON, suggesting that this process is sensitive to post-exercise nutrition (Fig. 9).

## Discussion

The protein complex mTORC1 is critical for regulating skeletal muscle mass^23^. Numerous studies in humans have demonstrated that mTORC1 activity is increased during the post-exercise period both with and without nutrient (e.g. amino acids) ingestion^1^. Inhibition of mTORC1 activity using the mTORC1 specific inhibitor rapamycin blocks resistance-exercise mediated increases in protein synthesis, indicating that mTORC1 activity is required to activate muscle protein synthesis in humans^4, 5^. Despite this body of work, additional research is warranted to better understand how mTORC1 is activated in human skeletal muscle in response to anabolic stimuli such as resistance exercise (both with and without nutrient ingestion) and how mTORC1 activates pathways regulating mRNA translation and, hence, muscle protein synthesis^24^. To this end, utilising immunofluorescence approaches in human skeletal muscle we have detailed, for the first time, intracellular mTOR localisation and protein complex co-localisation in response to an anabolic stimulus. We demonstrate that mTOR co-localises with the lysosome in basal conditions and that these complexes translocate to the cell periphery, in close proximity to capillaries, in response to resistance exercise. In parallel, we also observed that resistance exercise stimulates TSC2 dissociation from Rheb and that this leads to a reduction in TSC2 abundance at the cell membrane, which would be consistent with enhanced mTOR kinase activity. Although post-exercise ingestion of PRO/CHO had little effect on mTOR translocation or TSC2-Rheb-mTOR association, nutrient ingestion did increase mTOR and eIF3F association post exercise; this interaction may contribute to greater S6K1 kinase activity in the present study and the previously well-documented enhancement of muscle protein synthesis after exercise in the fed state^11, 12^.

Recent work from the Hornberger lab^19^ in mice has suggested that recruitment of the catalytic component of mTORC1 (mTOR) to the late lysosomal membrane is an important event initiating the protein synthetic response. We therefore sought to utilise immunofluorescence approaches to detail this event in human skeletal muscle in response to resistance exercise during a post-exercise period in which rates of muscle protein synthesis are known to be elevated^7, 8^. Additionally, we investigated whether consumption of a protein/carbohydrate beverage post exercise, which would further enhance the muscle protein synthetic response^14, 25, 26^, would augment the mTOR compartmentalisation during recovery compared to an energy-free control. In contrast to cell and rodent studies, we observed co-localisation of mTOR with the lysosome in basal conditions, an interaction that is retained during the 3 h post-exercise recovery period independently of concomitant protein/carbohydrate ingestion. Thus, recruitment of mTOR to the late-lysosome compartment does not appear to be enhanced relative to basal conditions in response to anabolic stimuli such as amino acids and/or muscle contraction in human skeletal muscle, which is in contrast to reports in cell^15^ and rodent^19^ models. The explanation for the discrepancy between the present and previous data is not readily apparent; however, differences in nutrient availability in human skeletal muscle compared to cell-based models may play a role. For example, dissociation of mTOR from the lysosome in cell-based models has been observed in amino acid free conditions^15^, which is a scenario that would be more reflective of chronic fasting that could initiate an autophagic response^27^. Interestingly, in the same *in vitro* model, mild amino acid reduction does not result in mTOR dissociation from the lysosome and therefore mTOR activity is unaltered^27^. Similar results could also be expected in human muscle given that muscle protein breakdown (as part of the normal ‘turnover’ of muscle protein pools) functions to replenish the intracellular amino acid pool to support rates of basal muscle protein synthesis^28^, as such, even in the fasted state there may be sufficient intracellular amino acids to maintain mTOR localization with the lysosomal membrane. Thus, constant mTOR association with the lysosome may reflect amino acid availability and the requirement of mTOR to maintain basal protein synthesis in resting human skeletal muscle.

Our initial hypothesis was that mTOR translocation to the lysosome would be the key activation event for mTOR in response to an exercise stimulus. In contrast, our data demonstrate that mTOR/LAMP2 translocation to the cell periphery is a principal event relocating mTOR following resistance exercise. The direct physiological relevance of this event is currently unclear, however it has previously been suggested that lysosome migration to the cell periphery can act as a stimulus for mTORC1 activation^27^. For example, overexpression of two kinesin superfamily members (KIF1Bβ and KIF2) that target lysosomes to the cell periphery increased mTORC1 activity^27^. In contrast, siRNA-mediated knockdown of KIF1Bβ and KIF2 targeted lysosomes to peri-nuclear regions, which subsequently reduced mTORC1 activity and initiated autophagy^27^. As such, lysosomal distribution would appear to be a key initial event in the increased activity of mTOR in response to nutrient stimulation. It is possible that the exercise-induced mTOR complex migration reported herein may also function to situate mTOR adjacent to ribosomal RNA^29^, the ribosomal pool^30, 31^ and ribosomal associated proteins^32^ reported to localise in this region. By doing so, this translocation event would position mTOR in close proximity to its target substrates and associated proteins required for protein synthesis and translation initiation. Support for this hypothesis can be observed in the elegant work of Goodman and colleagues who utilised the SUnSET technique to demonstrate that protein synthesis in mature skeletal muscle fibres *in vivo* occurs predominantly at the plasma membrane with a more diffuse signal in the intermyofibrillar region^32, 33^. When coupled with our data regarding mTOR migration and the known peripheral localization of the myonuclei and associated endoplasmic reticula, these studies collectively support a model in which the cell periphery is an active hub to support mRNA translation and protein synthesis in skeletal muscle. Our finding of a similar mTOR/LAMP2 translocation event following resistance exercise that is uninfluenced by protein/carbohydrate ingestion would suggest that mTOR/LAMP2 translocation is important for the initial activation of mRNA translation after exercise in human skeletal muscle.

Previous studies have demonstrated that mTOR interaction with GTP bound Rheb is required to elicit complete mTORC1 activation^34^. Rheb is a small GTPase^34^ and is activated by the GTPase activating protein TSC2, which converts GTP-Rheb into GDP-Rheb rendering it unable to activate mTORC1^18, 35^. Rheb is able to bind to the catalytic domain of mTOR^36, 37^ only when associated with GTP^38^. It was recently reported that eccentric contraction in mouse skeletal muscle leads to TSC2 dissociation from the lysosome, which would facilitate the mTOR and GTP-Rheb interaction^19^. In contrast, we observed TSC2 to be located at the plasma membrane, co-localising with Rheb as opposed to the two proteins associating at the lysosome. In our hands, TSC2 dissociated from the membrane following resistance exercise, leaving Rheb and the mTOR/LAMP2 complex to co-localise. It is unclear why the location of this event is different in human as opposed to murine skeletal muscle but may be reflective of muscle fibre-type differences (predominantly fast-twitch in mouse vs mixed fibres in human), the mode of exercise (eccentric vs concentric/eccentric), the degree of contraction or fibre recruitment, the nutrient availability in each scenario, and/or interspecies differences. Irrespective, our data are in agreement with Jacobs *et al*. reporting that TSC2 dissociation from mTOR positive regions^19^ and appears to be a key post-exercise event following an anabolic stimulus in human skeletal muscle. TSC2 dissociation from Rheb is thought to occur primarily via AKT-mediated phosphorylation^16, 39^. In support, we observed AKT kinase activity to be rapidly increased in skeletal muscle in both groups, peaking 1 h post exercise. This signalling was concomitant with dissociation of TSC2 from Rheb and a reduction in TSC2 at the cell membrane. However, there was no difference in TCS2/Rheb colocalization (or WGA-associated TSC2 signal) between the CON and FED groups at 1 h despite the large difference in AKT and S6K1 activity. This discrepancy could be related to a dissociated anabolic signalling response^40^, a greater methodological sensitivity for the activity rather than action (e.g. TSC2/Rheb dissociation) of AKT (i.e. kinase assay vs. immunofluorescence), and/or indicate that alternative kinases may regulate TSC2 activity in skeletal muscle, as has recently been proposed^22, 41^.

In addition to TSC2-Rheb-mTOR co-localisation, we next sought to examine whether mTOR translocation to the plasma membrane may serve to direct mTOR/LAMP2 to other substrates involved in the initiation of protein synthesis. We report novel data indicating that mTOR associates with the regulatory subunit of the eukaryotic initiation factor 3 complex, eIF3F, post-exercise in both the CON and FED groups. The eIF3 complex is key to the initiation of protein synthesis serving as a scaffold for mTOR and S6K1 interaction, leading to the assembly of the pre-initiation complex^42, 43^. mTOR and eIF3F co-localisation occurred rapidly post-exercise in both the CON and FED groups, which is consistent with the ability of resistance exercise to enhance amino acid uptake and muscle protein synthesis early in recovery^7, 44^. However this association was significantly greater in the FED group 1 h post exercise, suggesting that feeding may enhance mTOR-eIF3F assembly post-exercise. Given that muscle protein synthesis and the phosphorylation of mTORC1 substrates is amplified post-exercise following protein/carbohydrate ingestion^14, 26^, it would appear that the increased mTOR-eIF3F interaction observed in the FED group may be an important mediator of post-exercise nutrition on mTORC1 dependent signaling and potentially muscle protein synthesis. In support, both upstream (AKT) and downstream (S6K1) mTORC1-dependent kinase activity was enhanced by feeding in the present study, which would be in agreement with previous observations^45^ and consistent with a greater translational activity with protein/carbohydrate ingestion.

In addition, we also observed that mTOR/LAMP2 complexes associated with the microvasculature (UAE-I positive regions) at the plasma membrane post exercise. The precise reason for this response is currently unclear, however mTOR association with the microvasculature would in theory position mTOR in close proximity to nutrient provision delivered from the bloodstream. mTOR interaction with UEA-I positive cells increased equally post-exercise in both the CON and FED groups suggesting that mTOR interaction with the microvasculature is not influenced by protein/carbohydrate ingestion in healthy young men after exercise. Collectively, this adds further support to the hypothesis that mTOR translocation is a mechanically driven event and that protein/carbohydrate facilitates mTOR interaction with protein partners (eIF3F) rather than altering the localisation of mTOR/LAMP2 complexes.

### Limitations and technical considerations

The present study is the first to examine mTOR protein complex co-localisation in human skeletal muscle using immunofluorescence approaches. This novel approach provides a powerful tool to examine the relevance of cellular distribution and protein interaction for mTORC1 function, data currently lacking in the field. However, we would be remiss if we did not highlight that this approach relies heavily on immunofluorescence approaches, and by extension the specificity of the antibodies used. In an attempt to add confidence to our data and validate the specificity of each antibody, we performed competition assays with antigen specific peptides/recombinant proteins and used skeletal muscle from mTOR, Rheb and TSC2 knockout mice^21, 22^. These assays demonstrated specificity of the antibodies to their protein targets (i.e the signal was reduced in mKO vs WT), however they did also highlight non-specific binding that may have influenced our data. For example, use of mKO mouse tissue lead to an ~50% reduction the signal intensity of mTOR, TSC2 and Rheb, suggesting that the remaining signal could be attributed to non-specific binding of the antibodies used. Extending this observation into human skeletal muscle is challenging given potential species cross-reactivity of each antibody, histological differences in mouse vs human skeletal muscle (i.e fibre-type variation), in addition to contamination from other cell types. We attempted to bridge this gap by performing peptide/recombinant protein competition assays in human skeletal muscle. This approach was successful in blocking antibody binding to its target protein, however again cannot directly address the issue of non-specific binding. Collectively, therefore, whilst the approaches we used are considered ‘gold standard’ for the field, interpretative caution to our data should be employed given the potential for non-specific biding to have occurred, thus influencing our results. Whilst non-specific binding is a clear limitation of immunofluorescence approaches, the goal of this present study was to conduct a novel investigation into the localisation of mTOR and associated proteins, a task impossible using other analytical techniques. Therefore, despite the inherent limitations of immunofluorescent labelling in human skeletal muscle, we believe confidence can be taken from our results given our data shows mTOR and mTOR-related proteins to localize in specific patterns within the cell, interact with characterized protein partners, and translocate in a time-dependent manner in response to a physiological stimulus, all consistent with our current understanding of mTOR biology.

In summary, utilising immunofluorescence approaches in human skeletal muscle, we have detailed for the first time intracellular mTOR localisation and protein complex co-localisation in response to an anabolic stimulus. We demonstrated that mTOR co-localised with the lysosome in basal conditions and that these complexes translocated to the cell periphery in response to resistance exercise. In parallel, resistance exercise stimulates TSC2 dissociation from Rheb and leads to a reduction in TSC2 abundance at the cell membrane, which would be consistent with an enhanced mTOR kinase activity. Although post-exercise ingestion of protein/carbohydrate had little effect on mTOR translocation or TSC2-Rheb-mTOR co-localisation, nutrient ingestion did increase mTOR and eIF3F association as well as S6K1 kinase activity post exercise; an effect that may contribute to the well-documented enhancement of muscle protein synthesis after exercise in the fed state. Collectively, our data highlight the potential importance of mTOR-lysosomal cellular partitioning for mTORC1 function following a growth stimulus and illustrate another level of complexity to the molecular control of protein synthesis following resistance exercise and nutrition in human skeletal muscle. Future studies could include concurrent measures of muscle protein synthesis and effector enzyme activity^45^ to further elucidate the physiological significance of mTORC1 complex assembly and intracellular translocation in human skeletal muscle after exercise.

## Methods

### Subjects

Fourteen healthy, recreationally active males volunteered to participate in the study. Participants were informed about the experimental procedure to be used as well as the purpose of the study and all potential risks prior to obtaining written consent. All participants were deemed healthy based on their response to a routine medical screening questionnaire. All participants were recreationally active and reported performing resistance exercise with their lower body at least once per week. The study carried approval by the local Research Ethics Board of McMaster University and Hamilton Health Sciences and the University of Guelph Research Ethics Board and conformed to all standards for the use of human subjects in research as outlined in the Declaration of Helsinki.

### Experimental Design

Participants were randomly assigned into groups that received either a protein-carbohydrate beverage (FED; n = 7, age = 25 ± 2 y, BMI = 26.1 ± 2.3 kg/m^2^; means ± SD) or an energy-free control (CON; n = 7, age = 24 ± 3 y, BMI = 26.3 ± 2.6 kg/m^2^) after exercise. Participants reported to the laboratory after an overnight fast having refrained from strenuous exercise for at least 48 h. A single biopsy was taken from the vastus lateralis of a randomly selected thigh under local anesthesia (2% xylocaine), as previously described^46^. Participants performed an intense bout of bilateral leg resistance exercise consisting of 5 sets each of leg press and knee extension (with an inter-set rest period of 2-min) using a predetermined weight that elicited voluntary failure in 8–10 repetitions. Similar exercise protocols have been shown to elicit a robust increase in muscle protein synthesis over 3 h of recovery in both the fasted^47^ and fed states^25^. A second muscle biopsy was taken 10 min after completion of the exercise bout from the same thigh as the first biopsy to determine mTOR co-localization early in recovery. Participants were then randomly assigned to consume a commercially available beverage (Gatorade Recover®, Gatorade, IL, USA) providing 20/44/1 g of protein/carbohydrate/fat (FED) or an energy-free control (CON). As such, the 20 g of high quality milk-based protein consumed by FED would be expected to maximize muscle protein synthesis^48^ and the 44 g of carbohydrate would elicit a marked insulin response^26^ to enhance mTOR signalling^40^. Subsequently, muscle biopsies were taken 1 and 3 h after the beverage ingestion from separate incisions on the contralateral thigh from the initial biopsies to determine the time-dependent changes in mTOR co-localization during recovery in CON and FED.

### Skeletal muscle immunohistochemistry

Biopsy samples (~25 mg) were blotted and freed from any visible fat and connective tissue prior to being mounted in Optimal Cutting Temperature Compound (Tissue-Tek®, VWR) and frozen in isopentane cooled by liquid nitrogen prior to storage at −80 °C for subsequent immunofluorescence analysis. Embedded muscle samples were fixed on the position in front of the blade of the microtome (Bright 5040, Bright Instrument Company limited, Huntingdon, England) and serial sections (5 μm) collected onto room temperature uncoated glass slides (VWR international, UK). Sections were left to air dry at room temperature for 10 min to remove excess crystallized water inside sections under storage.

Sections (5 μm) were fixed in acetone and ethanol (3:1) solution (Fisher technology) for 5 min and then washed for 3 × 5 min in phosphate buffered saline (PBS) to remove fixation reagent. Sections were subsequently pre-incubated with 5% normal goat serum for 30 min, the PBS wash step repeated, prior to incubation (2 h) in primary antibody solution diluted with 5% normal goat serum (Invitrogen, UK). Following incubation, sections were washed for 3 × 5 min in PBS and incubated in the appropriate secondary antibody for 30 min at room temperature. Sections were finally incubated with wheat Germ Agglutinin (WGA-350) for 20 min at room temperature to mark the sarcolemmal membrane. Slides were left to air dry until the visual water stains evaporated for 1–2 min at room temperature. Then sections were mounted with 20 µL Mowiol® 4–88 (Sigma-Aldrich, UK) and sealed by glass coverslips to protect the muscle sections and to preserve fluorescence signals. Slides were left overnight before observation. All primary antibodies, corresponding secondary antibodies and working dilutions are listed in Table 1.

**Table 1:** List of primary and corresponding secondary antibodies used throughout the study.

Primary Antibody	Source	Dilution	Secondary Antibody
Monoclonal anti- mTOR antibody with mouse antigen, isotype IgG γ1 kappa	Millipore, 05-1592	1 in 200	Goat anti- mouse IgG γ1 Alexa®594
Polyclonal anti- Lamp2 antibody with rabbit antigen, isotype IgG	Abgent, AP1824d	1 in 100	Goat anti- rabbit IgG(H + L) Alexa®488
Monoclonal anti- dystrophin antibody with mouse antigen, isotype IgG 2α	Iowa Hybridoma Repository MANDYS1 clone 3B7	1 in 200	Goat anti- mouse IgG 2α Alexa®488
Monoclonal anti- Rheb antibody with rabbit antigen, isotype IgG	AbCam, ab92313	1 in 50	Goat anti- rabbit IgG (H + L) Alexa®488
Polyclonal anti- eIF3F antibody with rabbit antigen, isotype IgG	AbCam, ab74568	1 in 150	Goat anti- rabbit IgG (H + L) Alexa®488
Monoclonal anti- Tuberin antibody with mouse antigen, isotype IgGγ1	AM1919b	1 in 50	Goat anti- mouse IgG γ1 Alexa®594
Lectin from Ulex europaeus Agglutinin conjugated with FITC fluorescences (UEA-I)	Sigma Aldrich, L9006	1 in 300	Alexa Fluor® 488 Conjugated
Wheat Germ Agglutinin-350	W11263, Invitrogen	1 in 20	Alexa Fluor® 350 Conjugated

Antibody information.

### Antibody validation experiments

To confirm antibody specificity we employed antigen competition assays using synthetic peptides (Pepceuticals, Ltd, UK) generated against Rheb (QFVDSYDPTIENTFTKLITVNGQEYHLQLVDTAGQDEYSIFPQTYSIDING) and against eIF3f (CVDSYERRNEGAARV). This approach was not appropriate for TSC2 given that a fusion protein was used for antibody synthesis. Therefore we employed the recombinant TSC2 protein used in antibody generation (kindly provided by Abbgent, USA). In each case, the antibody was incubated with saturating concentrations (10 times higher than the antibody concentration) of the antigen peptide (Rheb, eIF3F) or recombinant protein (TSC2) for 24 h at 4 °C, prior to application onto human skeletal muscle sections in place of primary antibody. All other stages of the staining protocol were performed as described previously, with competition staining performed alongside a negative control in which the primary antibody was omitted completely. For mTOR, Rheb and TSC2 antibody specificity experiments, skeletal muscle cross-sections were obtained from the Tibialis Anterior muscle of mTOR conditional knock-out mouse samples or ‘floxed’ littermate controls^21^, or plantaris muscle from tamoxifen-inducible, muscle-specific iRheb and iTSC2 knockout mice compared to sham controls^22^. Rheb and TSC2 imKO samples were generated as previously described^22^. Briefly, animals received an initial 5-day treatment of Tamoxifen (2 mg/day), followed by 14 days without treatment, and then finally by a second round of Tamoxifen (1 mg/day) administration for 14 days. Samples were visualized as previously described for human samples.

### Image capture

Prepared slides were observed under a Nikon E600 microscope using a 40 × 0.75 numerical aperture objective. Images per area were captured under three colour filters achieved by a SPOT RT KE colour three shot CCD camera (Diagnostic Instruments Inc., MI, USA), illuminated by a 170 W Xenon light source. For images capture, DAPI UV (340–380 nm) filter was used to view WGA-350 (blue) signals and mTOR stains tagged with Alexa 594 fluorophore (red) was visualised under the Texas red (540–580 nm) excitation filter. FITC (465–495 nm) excitation filter was left to capture signals of mTOR-associated proteins, which were conjugated with Alexa Fluor 488 fluorophore unless stated otherwise (Table 1). DAPI UV (340–380 nm) was also used to observe the DAPI stained nucleus. All widefield images were obtained using a 40x objective (0.75 NA). Co-localisation experiments between mTOR and associated proteins were performed on an upright confocal microscope (Zeiss LSM 510 Meta, Carl Zeiss), using a 40 × 1.4 NA water immersion objective. Fluorophores were visualised under three laser filters simultaneously. Alexa Fluor 488 fluorophore was excited by an argon laser and 498–571 nm emission, while a 594 nm line of the helium–neon laser with 601–713 nm emission was used to excite Alexa Fluor 594 fluorophore. WGA conjugated with Alexa 350 and DAPI could be visualised under the excitation of the 405 nm line from a Diode 405–30 filter.

For total protein quantification, 3 slide replicates for each individual were stained, imaged and quantified. At least 7 images were captured per section with each image including ~6 fibres on average. For each subject, muscle samples were taken from four time points, and averaged values were calculated from two sections (repeats) for each time point. As such, the total fibre numbers analyzed for each subject was ~240–336 fibres.

All image processing and quantitation was carried out in ImagePro Plus 5.1 and kept constant between images, including exposure time, gain, and image frame. Total protein fluorescence intensity was quantified by measuring the signal intensity within the intracellular regions of a mask created by a specific membrane marker (dystrophin, WGA). For colocalisation analysis, five to seven areas per section were randomly selected and imaged under the same capture settings. Images were processed and analysed under the Image-Pro Plus 5.1 software (Media Cybernetics, MD, USA). Prior to colocalisation analysis, all images underwent a no neighbour deconvolution algorithm as a filter. Image signals generated by WGA or dystrophin were used to estimate cell membrane borders, which were merged with the corresponding target protein images to identify the association between the protein of interest and the plasma membrane. Pearson’s correlation coefficient (Image-Pro software) was used to quantify localization with the plasma membrane and mTOR associated proteins.

### S6K1 and AKT activity assays

50 mg of muscle was used for the measurement of S6K1 and AKT activity as previously described^45^.

### Statistical Analysis

Immunofluorescence image analysis was performed in duplicate, with 5–7 regions per cross section used for analysis. Pearson’s correlation coefficient values at each group/time point were analyzed using a two-way ANOVA (Prism, Graphpad) with Tukeys post-hoc analysis where appropriate. Significance was set at *P* < 0.05. Data are presented as Mean ± SEM and reported relative to the baseline biopsy (i.e. −40 min timepoint) in the CON group (PRE-EX CON).
